# Comparative assessment of small airway dysfunction by impulse oscillometry and spirometry in chronic obstructive pulmonary disease and asthma with and without fixed airflow obstruction

**DOI:** 10.3389/fmed.2023.1181188

**Published:** 2023-05-17

**Authors:** Chalerm Liwsrisakun, Warawut Chaiwong, Chaicharn Pothirat

**Affiliations:** Division of Pulmonary, Critical Care, and Allergy, Department of Internal Medicine, Faculty of Medicine, Chiang Mai University, Chiang Mai, Thailand

**Keywords:** COPD, asthma, fixed airflow obstruction, impulse oscillometry, small airways, spirometry

## Abstract

**Background:**

Small airways play a major role in the pathogenesis and prognosis of chronic obstructive pulmonary disease (COPD) and asthma. More data on small airway dysfunction (SAD) using spirometry and impulse oscillometry (IOS) in these populations are required. The objective of this study was to compare the two methods, spirometry and IOS, for SAD detection and its prevalence defined by spirometry and IOS in subjects with COPD and asthma with and without fixed airflow obstruction (FAO).

**Design:**

This is a cross-sectional study.

**Methods:**

Spirometric and IOS parameters were compared across four groups (COPD, asthma with FAO, asthma without FAO, and healthy subjects). SAD defined by spirometry and IOS criteria were compared.

**Results:**

A total of 262 subjects (67 COPD, 55 asthma with FAO, 101 asthma without FAO, and 39 healthy controls) were included. The prevalence of SAD defined by using IOS and spirometry criteria was significantly higher in patients with COPD (62.7 and 95.5%), asthma with FAO (63.6 and 98.2%), and asthma without FAO (38.6 and 19.8%) in comparison with healthy control (7.7 and 2.6%). IOS is more sensitive than spirometry in the detection of SAD in asthma without FAO (38.6% vs. 19.8%, *p* = 0.003) However, in subjects with FAO (COPD and asthma with FAO), spirometry is more sensitive than IOS to detect SAD (95.5% vs. 62.7%, *p* < 0.001 and 98.2% vs. 63.6%, *p* < 0.001, respectively).

**Conclusion:**

Small airway dysfunction was significantly detected in COPD and asthma with and without FAO. Although IOS shows more sensitivity than spirometry in the detection of SAD in asthma without FAO, spirometry is more sensitive than IOS in patients with FAO including COPD and asthma with FAO.

## Introduction

Small airways have a diameter of fewer than 2 mm (1). Small airways contribute a major role in both chronic obstructive pulmonary disease (COPD) and asthma (2, 3) caused by inflammation, hypersecretion of mucus, and airway remodeling (4). Small airway dysfunction (SAD) has been investigated for more than 60 years (5). No gold standard method currently exists for clinically assessing SAD. Spirometry is the most widely used method due to its relatively easy performance and simple measurement device (6).

The mid-maximal expiratory flow rate (MMEF) widely known as the average expired flow over the middle half (25–75%) of the forced vital capacity (FVC) maneuver (FEF25–75%) was proposed as the best parameter to identify SAD by spirometry (7). Its use is based on the hypothesis that the mid-late portion of the FVC reflects the airflow through the small airways, which are prone to expiratory collapse due to their lack of cartilaginous support (8). The American Thoracic Society (ATS) and European Respiratory Society (ERS) guidelines do not support using the FEF25–75% to identify SAD but suggest that oscillometry may provide evidence of airflow obstruction (9–11). Moreover, Quanjer et al. also suggested that the FEF25–75% does not contribute usefully to clinical decision-making (12). However, a recent large cohort analysis supports %predicted of FEF25–75% which helps link the anatomic pathology and deranged physiology of COPD independent of the %predicted forced expiratory measurement in the first second (FEV_1_) (13).

Impulse oscillometry (IOS) is a simple, non-invasive method, requiring only tidal breathing that allows for the evaluation of airway resistance and airway reactance in the airways and lungs (14). The IOS can be used for the diagnosis of COPD (15). It has been valuable for the evaluation of asthma control (16–18). Moreover, it detects SAD in COPD and asthma (19–22). Additionally, the IOS helped in the differentiation of COPD, asthma, and healthy subjects (15, 21, 23). All of the IOS parameters including resistance at 5 Hz (R5), resistance at 20 Hz (R20), heterogeneity of resistance (R5-R20), reactance at 5 Hz (X5), resonant frequency (Fres), and area under reactance curve between 5 Hz and Fres (AX) were significantly higher in COPD compared to healthy subjects (15, 23). The R20, R5–R20, and Fres were significantly different between COPD and asthma (23). Pornsuriyasak et al. also found significantly different IOS parameters across COPD, asthma with fixed airflow obstruction (FAO), and asthma without FAO (21).

In COPD and asthma, the small airway plays a major role in their pathogenesis (23). Air trapping and small airway wall thickening are associated with the progression of COPD (23). In asthma, inflammation and alterations of the small airways are associated with the severity of asthma and the level of asthma control (18, 24). Many methods have been introduced for measuring SAD including spirometry, plethysmography, IOS, inert gas washout, exhaled nitric oxide, and imaging, e.g., high-resolution computed tomography hyperpolarized magnetic resonance imaging and nuclear medicine (25). Of these, IOS is non-invasive, easy to perform, effort independent, and reproducible (25). By using IOS, more than half of subjects with COPD (60.0–74.0%) had SAD (21, 26–28). Additionally, Crisafulli et al. found the presence of SAD to progressively increase according to severity classifications of COPD (26). In asthma, a systematic review showed that the prevalence of SAD in asthma ranged from 33.0 to 69.9% when using IOS (22). In one study, the prevalence of SAD by using fixed R5–R20 criteria ≥0.075 kPa/L/s in COPD, asthma with FAO, and asthma without FAO were 68, 95, and 77%, respectively (21). By using spirometry, Manoharan et al. found that 54% of asthma had SAD defined using FEF25–75% of <60% predicted (29). The prevalence of SAD in airway diseases utilizing different methods varies. The study of SAD in COPD and asthma prevalence using IOS and spirometry still requires more clarification. Therefore, the objective of this study was to compare the IOS parameters and prevalence of SAD using IOS and spirometry in subjects with COPD, asthma with and without FAO, and healthy controls.

## Materials and methods

### Study procedures

This is a secondary analysis of cross-sectional studies in patients with COPD and asthma which were previously published (15, 18). The study was conducted at the Lung Health Center, Division of Pulmonary, Critical Care and Allergy, Department of Internal Medicine, Faculty of Medicine, Chiang Mai University, Chiang Mai, Thailand. All subjects were enrolled between July 2019 and June 2020. All tests were performed by a well-trained technician. Demographic data including age, sex, body mass index (BMI), comorbidities, smoking status, and inhaled medication used were recorded. The study was approved by the Research Ethics Committee of the Faculty of Medicine, Chiang Mai University [Institutional Review Board (IRB) approval number: MED-2562-06282, date of approval: 28 June 2019 and filed under Clinical Trials Registry (Study ID: TCTR20190709004, date of approval: 5 July 2019)]. Written informed consent was obtained from all subjects before enrollment.

### Subjects

The inclusion criteria were subjects with ages greater than 40 years. The exclusion criteria were the subjects who were unable to perform acceptable spirometry according to the ATS/ERS standard (11) and who were unable to perform the IOS according to the standard recommended by the ERS standard (14). Diagnosis of COPD in this study was based on the post-bronchodilator (post-BD) ratio of FEV_1_/FVC below the lower limit of normal (LLN) (9) with a history of smoking ≥10 pack-years, age of onset of symptoms >40 years, and no history of asthma in first-degree family members. Asthma subjects in this study were based on a history of clinically diagnosed asthma based on episodic breathlessness, wheezing, cough, and tightness and/or bronchodilator responsiveness and had a history of non-smoking or ex-smoking of <5 pack-years. Asthma with FAO was defined by the on-treatment ratio of FEV_1_/FVC persistently below LLN for at least three measurements during stable visits over a year. Asthma without FAO was characterized by the on-treatment ratio of FEV_1_/FVC ≥ LLN measured at the stable visit. Healthy control subjects were classified as subjects with normal spirometry (%FEV_1_/FVC value > LLN and FVC > LLN), had no chronic respiratory symptoms, no previous diagnosis of any chronic respiratory diseases, and were non-smokers (30).

### Definition

Due to the lack of consensus over which spirometry parameter is the best to identify SAD. FEF25–75% is a more sensitive parameter than FEV_1_ for assessing changes in peripheral airway function (7). FEF25–75% has been described as less effort-dependent than FEV_1_ and is a measurement of SAD (7, 8). Thus, this study used FEF25–75% from spirometry to identify SAD. However, there is no guideline regarding normal values for FEF25–75%. The most popular arbitrary cutoffs of FEF25-75% were between 60 and 75% (8). The previous study found that the normal 95th percentile for FEF25-75% is actually closer to 56% predicted in subjects with ages over 36 years (31). Therefore, the FEF25–75% < 60% predicted (8) was defined as SAD in our study. For the IOS test, SAD was defined when R5–R20 > ULN [predictive value +1.645 × Root Mean Square Error (RMSE)] (32).

### Spirometry

Spirometry was performed using the Vmax 22 spirometer, Care Fusion, Hoechberg, Germany. Pre-BD spirometry was performed according to the standards of ATS/ERS (10). Spirometric parameters were collected including FVC, FEV_1_, the ratio of FEV_1_/FVC, and FEF25–75%. The predicted values of FVC, FEV_1_, and FEF25-75% were calculated using the Global Lung Initiative (GLI) 2012 (Southeast Asian sub-group) reference equations (33). The *z*-scores of FVC, FEV_1_, ratio of FEV_1_/FVC, and FEF25–75% of each subject were also calculated from the GLI 2012 (Southeast Asian sub-group) reference equations (33).

### Impulse oscillometry

Pre-BD IOS was performed in all subjects before spirometry. The respiratory resistance and reactance were measured using IOS (Master Screen IOS, Viasys GmbH, Hoechberg, Germany). Each subject was asked to perform tidal breathing for 30–40 s *via* a mouthpiece that was connected to the IOS machine. A minimum of three tests following the recommendation by ERS standard were required (34). The average values from three IOS measurements were recorded. We collected the following IOS parameters: R5, R20, R5–R20, X5, Fres, and AX. The predicted values of all parameters in IOS were calculated using the Thai predictive value published by Deesomchok et al. (35).

### Study size calculation

The size of this study was calculated based on data from the previous study (21) using G*Power Version 3.1.9.2. The proportions of SAD in patients with COPD and asthma with FAO were 68 and 95%, respectively (21). Therefore, at least 140 subjects (35 subjects for each group) needed to be included in this study (power = 0.8 with statistical significance <0.05).

### Statistical analysis

Results for continuous data were expressed as mean ± standard deviation (SD) or median, interquartile range (IQR) as appropriate. Results for categorical data were expressed as frequencies and percentages. For baseline characteristics, IOS, and spirometry parameters, one-way analysis of variance (ANOVA) with the Bonferroni adjustment method was used to analyze differences across the four groups for parametric data. Kruskall–Wallis test was used to analyze the differences in baseline characteristics, IOS, and spirometry parameters across the four groups for non-parametric data. The Mann–Whitney *U*-Test was used to compare differences between the two groups for non-parametric data. The chi-square and Fisher’s exact test were used to compare the categorical data across the four groups and between groups, respectively. The McNemar test was used to compare the categorical data within the group. The correlations between the IOS parameters and FEF25–75% were determined using Spearman’s correlation coefficient analysis. The following cutoff parameters: 0 < |*r*| < 0.3 = weak correlation; 0.3 < |*r*| < 0.7 = moderate correlation; and |*r*| > 0.7 = strong correlation were used in this study (36). A value of *p* of <0.05 was considered statistical significance. In multiple comparisons, the adjusted level of significance was estimated by dividing the level of significance by several comparisons of four groups. Therefore, the value of *p* for multiple comparisons (6 and 3) was set as 0.008 (0.05/6) and 0.017 (0.05/3), respectively. All statistical analyses were performed using STATA version 16 (StataCorp, College Station, TX, United States).

## Results

A total of 262 subjects (67 COPD, 55 asthma with FAO, 101 asthma without FAO, and 39 healthy controls) were included in the analysis. The baseline demographic data of all subjects in the four groups are shown in Table 1. There were significant differences in age, proportion of male sex, BMI, smoking status, and cardiovascular comorbidity across the four groups. Inhaled medications used were significantly different between asthma with FAO and asthma without FAO groups. Age and male sex were significantly higher in the COPD group in comparison with the other groups. More data are shown in Table 1.

**Table 1:** Baseline characteristics of all subjects.

Clinical characteristics	Newly diagnosed COPD (*n* = 67)	Asthma with FAO (*n* = 55)	Asthma without FAO (*n* = 101)	Healthy control (*n* = 39)	*p*-value
Age (year)	69.1 ± 8.5	62.9 ± 10.9*	60.8 ± 9.9*	60.6 ± 10.4*	<0.001
Male sex *n* (%)	59 (88.1)	23 (41.8)	31 (30.7)	21 (53.8)	<0.001
Body mass index (BMI)^a^	22.7 ± 4.4	24.6 ± 4.6*	26.3 ± 4.4*	23.6 ± 3.0^##^	<0.001
Smoking status					<0.001
Non-smoker	0 (0.0)	48 (87.3)	89 (88.1)	39 (100.0)	
Ex-smoker	63 (94.0)	7 (12.7)	11 (10.9)	0 (0.0)	
Current-smoker	4 (6.0)	0 (0.0)	1 (1.0)	0 (0.0)	
Smoking pack-year (median, IQR)	25.0 (16.4, 42.0)	0.0 (0.0, 0.0)*	0.0 (0.0, 0.0)*	0.0 (0.0, 0.0)*	<0.001
Comorbidity *n* (%)					
Cardiovascular disease	40 (59.7)	20 (36.4)	37 (36.6)	5 (12.8)	<0.001
Metabolic disease	13 (19.4)	6 (10.9)	16 (15.8)	0 (0.0)	0.438
Neuromuscular disease	6 (9.0)	2 (3.6)	4 (4.0)	0 (0.0)	0.300
Inhaled medication used^a^					<0.001
ICS	N.A.	0 (0.0)	10 (9.9)	N.A.	
ICS + LABA	N.A.	33 (60.0)	77 (76.2)	N.A.	
ICS + LABA + LAMA	N.A.	22 (40.0)	14 (13.9)	N.A.	

Data are mean ± standard deviation (SD) unless otherwise stated; ^a^*p*-value from exact test comparing asthma with fixed airflow obstruction and asthma with normal spirometry; *p < 0.013 compared with COPD; ^#^*p* < 0.013 compared with asthma with FAO; ^##^*p* < 0.013 compared with asthma without FAO; value of *p* of difference between the groups was significant with an adjusted level of significance (0.05/4 = 0.013). COPD, chronic obstructive pulmonary disease; IQR, interquartile range; ICS, inhaled corticosteroid; LABA, Long-acting beta2-agonists; LAMA long-acting muscarinic antagonist; NA, not assessment; FAO, fixed airway obstruction.

There were significantly lower %predicted and *z*-score of all parameters of spirometry in the COPD and asthma with FAO groups in contrast to asthma without FAO and healthy control groups. The %predicted and *z*-score of all parameters of spirometry in the COPD group were also significantly lower than asthma with FAO group, except for the *z*-score of FEV_1_/FVC and %predicted and *z*-score of FEF25–75%. In asthma without FAO, the *z*-score of FEV_1_/FVC and %predicted and *z*-score of FEF25–75% were significantly lower against the healthy controls. More details are shown in Table 2.

**Table 2:** Spirometric data of all subjects.

Spirometry data (Pre-bronchodilator)	Newly diagnosed COPD (*n* = 67)	Asthma with FAO (*n* = 55)	Asthma without FAO (*n* = 101)	Healthy control (*n* = 39)	*p*-value
%Predicted FVC	82.2 ± 18.8*	91.5 ± 15.5*	98.2 ± 14.3*^,#^	101.5 ± 11.0*^,#^	<0.001
*z*-Score of FVC	−1.1 ± 1.1	−0.6 ± 1.0*	−0.1 ± 0.9*^,#^	0.1 ± 0.7*^,#^	<0.001
%Predicted FEV_1_	59.9 ± 18.5	70.1 ± 15.1*	92.2 ± 13.8*^,#^	100.2 ± 11.6*^,#^	<0.001
*z*-Score of FEV_1_	−2.3 ± 1.0	−1.8 ± 0.9*	−0.5 ± 0.9*^,#^	0.0 ± 0.7*^,#^	<0.001
FEV_1_/FVC (%)^a^	57.4 ± 9.1	62.1 ± 6.9*	76.9 ± 4.7*^,#^	80.5 ± 5.2*^,#^	<0.001
*z*-Score of FEV_1_/FVC	−2.9 ± 1.1	−2.7 ± 0.8	−0.8 ± 0.6*^,#^	−0.1 ± 0.7*^,#,##^	<0.001
%Predicted FEF25–75%	31.7 ± 14.1	37.6 ± 11.3	80.0 ± 24.1*^,#^	106.3 ± 31.7*^,#,##^	<0.001
*z*-Score of FEF25–75%	−2.5 ± 0.8	−2.3 ± 0.6	−0.7 ± 0.8*^,#^	0.1 ± 0.9*^,#,##^	<0.001

Data are mean ± standard deviation (SD); *p < 0.017 compared with COPD. #*p* < 0.013 compared with asthma with FAO; ##*p* < 0.013 compared with asthma without FAO; value of *p* of difference between groups was significant with an adjusted level of significance (0.05/4 = 0.013). FVC, forced vital capacity; FEV_1_, forced expiratory volume in the first second; FEF25–75%, forced expiratory flow at 25–75% of FVC; FAO, fixed airway obstruction.

Impulse oscillometry parameters of all groups are shown in Table 3. The %predicted and the absolute values of IOS parameters including R5–R20, Fres, and AX were significantly lower in healthy controls in correlation with the other groups. The less negative reactance of X5 was also observed in the healthy control group. In the COPD and asthma with FAO groups, a significant increase in %predicted and the absolute value of IOS parameters, including R5–R20, Fres, and AX, was observed in comparison with asthma without FAO group. A significant decrease in %predicted of X5 was observed in COPD and asthma with FAO when contrasted with asthma without FAO group. The %predicted of AX was significantly higher in the group with COPD when compared with asthma in the FAO group. The %predicted and absolute values of R5-R20 were indifferent between COPD and asthma in the FAO group. More data are shown in Table 3.

**Table 3:** Impulse oscillometry (IOS) parameters of all subjects.

IOS parameters	Newly diagnosed COPD (*n* = 67)	Asthma with FAO (*n* = 55)	Asthma without FAO (*n* = 101)	Healthy control (*n* = 39)	*p*-value
R5 (cmH_2_O/L/s)	4.8 ± 1.6	5.1 ± 2.1	4.6 ± 1.5	3.5 ± 1.2*^,#,##^	<0.001
% Predicted R5	158.3 ± 59.2	128.1 ± 48.4*	104.9 ± 36.5*^,#^	97.7 ± 22.6*^,#^	<0.001
R20 (cmH_2_O/L/s)	3.1 ± 0.9	3.3 ± 1.2	3.4 ± 0.9	2.9 ± 0.9	0.027
% Predicted R20	121.3 ± 34.8	101.2 ± 39.2*	96.5 ± 34.8*	96.4 ± 18.5*	<0.001
R5-R20 (cmH_2_O/L/s) (median, IQR)	1.6 (0.9, 2.2)	1.3 (0.8, 2.3)	0.9 (0.4, 1.8)*^,#^	0.5 (0.3, 0.9)*^,#,##^	<0.001
% Predicted R5-R20 (median, IQR)	282.1 (171.8, 412.8)	270.2 (153.6, 545.1)	154.7 (87.7, 281.4)*^,#^	96.7 (51.3, 158.7)*^,#,##^	<0.001
X5 (cmH_2_O/L/s) (median, IQR)	−1.9 (−2.7, −1.3)	−1.6 (−2.7, −0.9)	−1.5 (−1.9, −0.9)*	−1.0 (−1.3, −0.5)*^,#,##^	0.001
% Predicted X5 (median, IQR)	189.9 (141.1, 279.8)	151.7 (87.9, 258.8)*	140.8 (94.2, 177.9)*	95.7 (59.9, 138.4)*^,#,##^	<0.001
Fres (Hz)	22.9 ± 5.5	21.4 ± 7.4*	17.5 ± 5.1*^,#^	12.9 ± 3.9*^,#,##^	<0.001
% Predicted Fres	169.3 ± 47.7	157.3 ± 60.6	121.5 ± 32.7*^,#^	96.9 ± 25.5*^,#,##^	<0.001
AX (cmH_2_O/L) (median, IQR)	17.2 (8.9, 26.1)	11.0 (6.7, 24.4)	7.1 (3.6, 14.1)*^,#^	3.3 (1.5, 4.9)*^,#,##^	<0.001
% Predicted AX (median, IQR)	459.7 (234.9, 779.0)	298.2 (185.5, 567.9)*	166.2 (98.3, 309.5)*^,#^	91.0 (42.5, 141.3)*^,#,##^	<0.001

Data are mean ± standard deviation (SD) or otherwise stated; *p < 0.017 compared with COPD; ^#^*p* < 0.013 compared with asthma with FAO; ^##^*p* < 0.013 compared with asthma without FAO; value of *p* of difference between groups was significant with an adjusted level of significance (0.05/4 = 0.013). R5, resistance at 5 Hz; R20, resistance at 20 Hz; R5-R20; heterogeneity of resistance between R5 and R20; Fres, resonant frequency; X5, reactance at 5 Hz; AX, the area under reactance curve between 5 Hz and resonant frequency; FAO, fixed airway obstruction.

Correlations between FEF25–75% and IOS parameters in subjects with chronic respiratory diseases and healthy controls are shown in Table 4. There was only a low-to-moderate correlation between some parameters of IOS and FEF25–75%, in which Fres in asthma with FAO showed the highest correlation. Correlations between FEF25–75% and IOS parameters in subjects with COPD, asthma with FAO, and asthma without FAO and healthy control with preserved FVC are also shown in Table 5. Most of the IOS parameters did not correlate with FEF25–75%. The highest correlation was shown in FEF25–75% and Fres in asthma with FAO, which was only a moderate correlation.

**Table 4:** Spearman correlation coefficients of FEF25–75% and IOS parameters in subjects with chronic respiratory diseases and healthy control.

	R5–R20	%R5–R20	AX	%AX	X5	%X5	Fres	%Fres
Chronic respiratory diseases (*n* = 223)								
Newly diagnosed COPD (*n* = 67)	−0.422*	−0.441*	−0.453*	−0.460*	0.426*	−0.340*	−0.472*	−0.508*
Asthma with FAO (*n* = 55)	−0.213	−0.222	−0.326*	−0.377*	0.205	−0.204	−0.505*	−0.536*
Asthma without FAO (*n* = 101)	−0.209*	−0.185	−0.222*	−0.241*	0.155	−0.179	−0.271*	−0.246*
Healthy control (*n* = 39)	−0.354*	−0.292	−0.149	−0.138	−0.059	−0.039	−0.237	−0.275

*p < 0.05. R5-R20, heterogeneity of resistance between R5 and R20; Fres, resonant frequency; X5, reactance at 5 Hz; AX, the area under reactance curve between 5 Hz and resonant frequency; FAO, fixed airway obstruction.

**Table 5:** Spearman correlation coefficients of FEF25–75% and IOS parameters in subjects with chronic respiratory diseases and healthy control with FVC ≥ 80% predicted.

	R5-R20	%R5-R20	AX	%AX	X5	%X5	Fres	%Fres
Chronic respiratory diseases (*n* = 173)								
Newly diagnosed COPD (*n* = 40)	−0.158	−0.171	−0.216	−0.216	0.223	−0.146	−0.058	−0.109
Asthma with FAO (*n* = 44)	−0.102	−0.171	−0.224	−0.273	0.134	−0.109	−0.369*	−0.481*
Asthma without FAO (*n* = 89)	−0.216*	−0.227*	−0.263*	−0.299*	0.196	−0.206	−0.266*	−0.282*
Healthy control (*n* = 37)	−0.305	−0.233	−0.085	−0.086	−0.128	0.008	−0.221	−0.276

*p < 0.05. R5-R20, heterogeneity of resistance between R5 and R20; Fres, resonant frequency; X5, reactance at 5 Hz; AX, the area under reactance curve between 5 Hz and resonant frequency; FAO, fixed airway obstruction.

The prevalence of SAD was significantly higher in COPD, asthma with FAO, and asthma without FAO in comparison with healthy control groups both in the whole and subgroups with preserved FVC (Tables 6 7). In COPD and asthma with FAO groups, the prevalence of SAD was also significantly higher than in asthma without FAO group. But there was no difference in the prevalence of SAD between COPD and asthma with FAO groups (Tables 6 7). In the case of airway obstruction including COPD and asthma with FAO, the FEF25–75% was more sensitive than R5–R20 for SAD diagnosis. But, R5–R20 was more sensitive than FEF25–75% for SAD detection in asthma without FAO. Additionally, the overestimation of SAD was observed when using the fixed criteria (R5-R20 ≥ 0.76 cmH_2_O) in asthma without FAO and healthy controls. More data are shown in Tables 6 7.

**Table 6:** Subjects with small airway dysfunction defined by R5–R20 ≥ upper limit of normal, R5–R20 ≥ 0.76 cmH_2_O, and %predicted FEF25–75% < 60.

Small airway dysfunction	Newly diagnosed COPD (*n* = 67)	Asthma with FAO (*n* = 55)	Asthma without FAO (*n* = 101)	Healthy control (*n* = 39)	*p*-value
R5-R20 ≥ ULN	42 (62.7)	35 (63.6)	39 (38.6)*^,#^	3 (7.7)*^,#,##^	<0.001
R5-R20 ≥ 0.76 cmH_2_O (0.075 kPa/L/s)	56 (83.6)^a^	42 (76.4)^a^	56 (55.4)*^,#,a^	12 (30.8)*^,#,##,a^	<0.001
%Predicted FEF25–75% < 60	64 (95.5)^a^	54 (98.2)^a,b^	20 (19.8)*^,#,a,b^	1 (2.6)*^,#,##,b^	<0.001

Data are *n* (%); *p < 0.008 compared with COPD; ^#^*p* < 0.008 compared with asthma with FAO; ^##^*p* < 0.008 compared with asthma without FAO; value of *p* of difference between groups was significant with an adjusted level of significance (0.05/6 = 0.008); ^a^p < 0.017 compared with R5–R20 ≥ ULN criteria; ^b^compared with R5–R20 ≥ 0.76 cmH_2_O criteria; for ^a,b^value of *p* of difference between group was significant with an adjusted level of significance (0.05/3 = 0.017). R5-R20, heterogeneity of resistance between R5 and R20; ULN, upper limit of normal; FAO, fixed airway obstruction.

**Table 7:** Subjects with small airway dysfunction defined by R5–R20 ≥ upper limit of normal, R5–R20 ≥ 0.76 cmH_2_O, and %predicted FEF25-75% < 60 in subjects with chronic respiratory diseases and healthy control with preserved FVC (FVC ≥ 80% predicted).

Small airway dysfunction	Newly diagnosed COPD (*n* = 40)	Asthma with FAO (*n* = 44)	Asthma without FAO (*n* = 89)	Healthy control (*n* = 37)	*p*-value
R5–R20 ≥ ULN	20 (50.0)	26 (59.1)	34 (38.2)	3 (8.1)*^,#,##^	<0.001
R5–R20 ≥ 0.76 cmH_2_O (0.075 kPa/L/s)	32 (80.0)^a^	32 (72.7)	47 (52.8)*^,a^	11 (29.7)*^,##,a^	<0.001
%Predicted FEF25–75% < 60	37 (92.5)^a^	43 (97.7)^a,b^	18 (20.2)*^,#,a,b^	1 (2.7)*^,#,##,b^	<0.001

Data are *n* (%); *p < 0.008 compared with COPD; ^#^*p* < 0.008 compared with asthma with FAO; ^##^*p* < 0.008 compared with asthma without FAO; value of *p* of difference between groups was significant with an adjusted level of significance; (0.05/6 = 0.008), ^a^*p* < 0.017 compared with R5–R20 ≥ ULN criteria; ^b^compared with R5–R20 ≥ 0.76 cmH_2_O criteria; for ^a,b^, value of *p* of difference between group was significant with an adjusted level of significance (0.05/3 = 0.017). R5–R20, heterogeneity of resistance between R5 and R20; ULN, upper limit of normal; FAO, fixed airway obstruction.

## Discussion

In this study, we found that the prevalence of SAD classified by an increase in small airway resistance (R5–R20 ≥ ULN) was significantly higher in COPD, asthma with FAO, and asthma without FAO compared to healthy controls. The spirometry-derived FEF25–75% was more sensitive than R5–R20 for SAD diagnosis in patients with COPD and asthma with FAO. But, the IOS-derived R5–R20 was more sensitive than FEF25–75% for SAD diagnosis in asthma without FAO. In addition, SAD was overdiagnosed when using the fixed criteria of R5–R20 ≥ 0.76 cmH_2_O in asthma without FAO and healthy controls. We also found that the IOS parameters, especially for R5–R20, X5, Fres, and AX, were significantly lower in healthy subjects compared to COPD and asthma with and without FAO.

Respiratory resistance is largely affected by airway caliber (34). The narrower and longer airways have higher airway resistance (34). Our study showed an increase in R5 and R5–R20 in COPD, asthma with FAO, and asthma without FAO compared to healthy controls. Additionally, the R5–R20 was significantly higher in COPD and asthma with FAO compared to asthma without FAO groups. Our results were supported by the previous studies showing that airway resistances were increased in COPD and asthma (with and without FAO) compared to healthy subjects (15, 21, 23). Moreover, our results were comparable to the previous finding indicating that R5–R20 was increased in asthma with FAO compared to asthma without FAO (21).

Respiratory reactance is comprised of both inertance and elastance (34). King et al. suggested that more negative reactance indicated greater elastance or stiffness (34) and this typically occurred in subjects with obstructive airway diseases (34). The previous studies showed a decrease in X5 and an increase in AX in subjects with COPD and asthma compared with healthy controls (15, 23). In asthma with FAO, a decrease in X5 and an increase in AX were also observed in contrast with asthma without FAO (21). Our study also demonstrated that the X5 and AX were significantly decreased and increased, respectively, in COPD, asthma with FAO, and asthma without FAO when compared to healthy subjects.

From the previous studies, the diagnosis of SAD could be made by using IOS parameters, especially the R5–R20 (21, 22, 26, 27, 37). They reported that the ranges of the prevalence of SAD in COPD and asthma varied from 60.0 to 73.8% and 33.0 to 95.0%, respectively. In this study, we used the ULN of R5–R20 value for the diagnosis of SAD. We found the prevalence of SAD in COPD, asthma with FAO, and asthma without FAO to be 62.7, 63.6, and 38.6%, respectively. Our results were comparable with the previous findings (21, 22, 26, 27, 37). However, the prevalence of SAD in asthma with FAO and without FAO in our study was lower than in the previous study published by Pornsuriyasak et al. (21). They found that the prevalence of SAD was 95 and 77% in asthma with FAO and asthma without FAO, respectively. These discrepancies were due to the difference in criteria used for the diagnosis of SAD. The average of ULN of R5–R20 in our study was 1.182 cmH_2_O/L/s (0.116 kPa/L/s) (data not shown) which was much higher than the fixed cutoff R5–R20 level of >0.075 kPa/L/s used by the previous study (21). This could lead to overestimating SAD when using the fixed cutoff R5–R20 criteria.

This study found FEF25–75% to be more sensitive than R5–R20 in the case of FAO (COPD and asthma with FAO). R5–R20 was more sensitive than FEF25–75% for SAD detection in groups with normal FEV_1_/FVC (asthma without FAO) and in subgroups with preserved FVC. Additionally, the overestimation of SAD was observed when using the fixed criteria (R5-R20 ≥ 0.76 cmH_2_O) in asthma without FAO and healthy controls. Our results were supported by a previous study indicating that IOS shows more sensitivity for evaluating SAD than spirometry in patients with normal lung function and spirometry showed more sensitivity than IOS to detect SAD using spirometry in subjects with abnormal lung function (32). The difference in techniques of both tests might explain these discrepant results. The sound waves were applied to measure airway resistance and airway reactance during tidal breathing in IOS, whereas the forced expiratory maneuvers might induce airway collapse which resulted in decreasing of FEF25–75% in spirometry (8, 38). Therefore, the ULN of R5-R20 from IOS is more sensitive than FEF25–75% from spirometry for the detection of SAD in subjects with normal FEV_1_/FVC and patients with obstructive airway disease with normal FVC.

Impulse oscillometry is currently the most specific and sensitive test for the detection of SAD (37). We found that subjects with chronic respiratory disease including COPD and asthma were in high-risk groups for SAD because small airways play a major role in the pathogenesis and prognosis of both COPD and asthma (18, 23, 24). For example, the SAD classified by value of R5–R20 ≥ 1 cmH_2_O/L/s was an accurate tool for the detection of uncontrolled asthma in our previous study (18). SAD diagnosed by IOS in patients with intermittent asthma not treated with controller medications, mostly had normal FEV_1_/FVC, was shown to predict the future of more symptoms, more use of rescue medication, poor asthma control, and severe exacerbations (39). In addition, serial R5–R20 measurements were beneficial in predicting the response to treatment (40). These findings confirm that IOS has benefits in both prognosis and management plans in asthma patients. Therefore, the findings of these and our studies suggest that clinicians should regularly perform IOS testing in patients with obstructive airway disease. The results of SAD measured by IOS may be useful for monitoring and treating these patients.

The strength of our study is that the healthy control group was included for comparison with COPD and asthma with and without the FAO group. We also did the subgroup analysis of those with preserved FVC. Moreover, the prediction equations of IOS parameters in the Thai population were used in this study. The %predicted of each IOS parameter was used for analysis. Thus, the diversities of age, sex, height, and weight that had impacts on airway resistance and reactance were minimized (35, 41, 42). We encourage using the ULN criteria of R5-R20 instead of the fixed criteria (R5–R20 ≥ 0.76 cmH_2_O) for reducing the over-or underestimation of SAD. However, this study has some limitations. First, only newly diagnosed COPD was included in this study. Thus, the results may not be generalized for treated COPD patients. Second, due to the small sample size of COPD, the prevalence of SAD according to the staging of COPD was not mentioned in this study. Third, due to the lack of consensus over the cutoff value of FEF25–75% for identifying SAD, the threshold of FEF 25–75% < 60% predicted was classified as SAD in the current study. Thus, the results may not be generalized for the other studies which used other cutoff points. Finally, due to the cross-sectional study design, the association between a continuous scale of lung function measurements (FEF25–75% and R5–R20) and clinical symptoms, exacerbations, and comorbidities were not mentioned in this study. Therefore, the effect of SAD on clinical symptoms and exacerbations should be addressed in future prospective studies.

## Conclusion

The IOS parameters, especially the R5–R20, can be used to differentiate healthy subjects from chronic airway diseases, including COPD and asthma with or without FAO. The ULN, rather than a fixed cutoff point, of R5–R20 should be used to identify SAD. The prevalence of SAD was significantly higher in COPD, asthma with FAO, and asthma without FAO in comparison with healthy control. Moreover, the prevalence of SAD was significantly higher in COPD and asthma with FAO than in asthma without FAO. IOS is more sensitive than spirometry for the detection of SAD in asthma without AFO; however, for patients with AFO including COPD and asthma with FAO, spirometry is more sensitive.

## Data availability statement

The raw data supporting the conclusions of this article will be made available by the authors, without undue reservation.

## Ethics statement

The studies involving human participants were reviewed and approved by the Research Ethics Committee of the Faculty of Medicine, Chiang Mai University [Institutional Review Board (IRB) approval number: MED-2562-06282, date of approval: 28 June 2019 and filed under Clinical Trials Registry (Study ID: TCTR20190709004, date of approval: 5 July 2019)]. The patients/participants provided their written informed consent to participate in this study.

## Author contributions

CL, WC, and CP: conceptualization, methodology, validation, investigation, resources, writing–review and editing, and visualization. WC: software, formal analysis, and data curation. CL and WC: writing–original draft preparation. CL and CP: supervision and project administration. WC and CP: funding acquisition. All authors have read and agreed to the published version of the manuscript.

## Funding

This study was funded by the Faculty of Medicine, Chiang Mai University Research Fund under grant no. 037/2563.

## Conflict of interest

The authors declare that the research was conducted in the absence of any commercial or financial relationships that could be construed as a potential conflict of interest.

## Publisher’s note

All claims expressed in this article are solely those of the authors and do not necessarily represent those of their affiliated organizations, or those of the publisher, the editors and the reviewers. Any product that may be evaluated in this article, or claim that may be made by its manufacturer, is not guaranteed or endorsed by the publisher.
